# A Five-lncRNAs Signature-Derived Risk Score Based on TCGA and CGGA for Glioblastoma: Potential Prospects for Treatment Evaluation and Prognostic Prediction

**DOI:** 10.3389/fonc.2020.590352

**Published:** 2020-12-17

**Authors:** Xuegang Niu, Jiangnan Sun, Lingyin Meng, Tao Fang, Tongshuo Zhang, Jipeng Jiang, Huanming Li

**Affiliations:** ^1^Department of Neurosurgery, Tianjin 4th Central Hospital, Tianjin, China; ^2^Department of Psychiatry, Characteristic Medical Center of the Chinese People’s Armed Police Force, Tianjin, China; ^3^Department of Urology, Tianjin Institute of Urology, The Second Hospital of Tianjin Medical University, Tianjin, China; ^4^Central Laboratory, Tianjin 4th Central Hospital, Tianjin, China; ^5^Department of Laboratory, Jiangsu Provincial Corps Hospital of Chinese People’s Armed Police Force, Yangzhou, China; ^6^Postgraduate School, Medical School of Chinese PLA, Beijing, China; ^7^Department of Thoracic Surgery, The First Medical Centre, Chinese PLA General Hospital, Beijing, China

**Keywords:** lncRNA, glioblastoma, prognosis, The Cancer Genome Atlas, Chinese Glioma Genome Atlas

## Abstract

Accumulating studies have confirmed the crucial role of long non-coding RNAs (ncRNAs) as favorable biomarkers for cancer diagnosis, therapy, and prognosis prediction. In our recent study, we established a robust model which is based on multi-gene signature to predict the therapeutic efficacy and prognosis in glioblastoma (GBM), based on Chinese Glioma Genome Atlas (CGGA) and The Cancer Genome Atlas (TCGA) databases. lncRNA-seq data of GBM from TCGA and CGGA datasets were used to identify differentially expressed genes (DEGs) compared to normal brain tissues. The DEGs were then used for survival analysis by univariate and multivariate COX regression. Then we established a risk score model, depending on the gene signature of multiple survival-associated DEGs. Subsequently, Kaplan-Meier analysis was used for estimating the prognostic and predictive role of the model. Gene set enrichment analysis (GSEA) was applied to investigate the potential pathways associated to high-risk score by the R package “cluster profile” and Wiki-pathway. And five survival associated lncRNAs of GBM were identified: LNC01545, WDR11-AS1, NDUFA6-DT, FRY-AS1, TBX5-AS1. Then the risk score model was established and shows a desirable function for predicting overall survival (OS) in the GBM patients, which means the high-risk score significantly correlated with lower OS both in TCGA and CGGA cohort. GSEA showed that the high-risk score was enriched with PI3K-Akt, VEGFA-VEGFR2, TGF-beta, Notch, T-Cell pathways. Collectively, the five-lncRNAs signature-derived risk score presented satisfactory efficacies in predicting the therapeutic efficacy and prognosis in GBM and will be significant for guiding therapeutic strategies and research direction for GBM.

## Introduction

As one of the most common malignant brain tumor (1, 2), the 5-year survival rate for glioblastoma (GBM) in patients is less than 5%. Although great progress has occurred in the field of chemotherapy, radiotherapy, and surgical resection, the prognosis for GBM patients is still poor (3–5). Long-non-coding RNA (lncRNA) have been a focus in recent years since they play a nonnegligible role in a variety of biological processes and exert significant influence in human diseases. Particularly, the abnormal expression of lncRNA is tightly connected to the occurrence, prognosis, and survival of patients with cancer (6, 7). Numerous studies have illustrated that certain lncRNAs are aberrantly expressed in GBM tissue, and many of them have been confirmed to be involved in tumor invasion, immune escape and radiation resistance. In recent years, specific lncRNAs were also identified as prognostic biomarkers and therapeutic targets for GBM, while some of them were proposed to be novel indicators for survival prediction in GBM patients.

So, can we create a model on the basis of a multiple-gene signature that can advance the effectiveness of treatment evaluation and prognostic prediction for GBM? In the present study, we collected high-throughput data in TCGA and CGGA databases which were generated by microarrays and next-generation sequencing, then we identified some survival-related differentially expressed genes (DEGs) by analyzing the data of lncRNA expression in GBM, subsequently, a risk model for treatment evaluation and prognostic prediction was established on the basis of the identified gene signature. In this study, five lncRNAs in GBM were revealed notably related with survival independently, and the high-risk score was enriched with items of signaling pathways for oncogenesis and tumor progression. The risk score model based on the five-lncRNAs signature exhibited satisfactory efficacies in predicting the therapeutic efficacy and prognosis in GBM.

## Materials and Methods

### Publicly Available Clinical Data Sets

RNA-seq (Illumina RNASeqV2, Level 3; Illumina, San Diego, CA) FPKM (fragments per kilobase of transcript per million mapped reads) of GBM were downloaded from The Cancer Genome Atlas (TCGA; https://cancergenome.nih.gov) on October 1, 2019 (The database was updated to January 25, 2018 at that time), including 167 samples. RNA-seq expression data from STAR+RSEM (Illumina Hiseq; mRNAseq_693) of Glioma including 140 GBM samples were downloaded from Chinese Glioma Genome Atlas (CGGA; http://www.cgga.org.cn/index.jsp) on October 1, 2019 (8–10).

### Statistical Analyses

All statistical analyses were performed by R. For the TCGA and CGGA dataset, we first converted them to the same gene symbols and selected the common lncRNA, performing log2(FPKM/RSEM+1) transformation. Then the univariate cox regression analysis was used to find the correlation between the expression level of each single gene and patients’ OS. The genes were filtered using a p-value below 0.05. Then, we overlapped the common genes with HR>1 or HR<1 in TCGA and CGGA. We got 3 HR>1 genes and 4 HR<1 genes to perform the multivariate cox regression analysis using the TCGA data set as the training cohort. And we got the risk score formula of the prognostic model. The resulting model according to the formula: Risk score =−1.153*LINC01545 −0.537*WDR11−AS1; − 0.799*NDUFA6−DT −1.558*FRY−AS1+0.681*TBX5−AS1. In the formula, every single lncRNA represented its expression value, the values of coefficients represented the relative contributions of the five lncRNAs in the multivariate cox regression analysis. TBX5-AS1 is a risk factor while the other four genes may be protective factors. In the calculation of risk score, the higher the value is, the higher the risk of the patient may be. The optimal cut-off value was used to calculate the high-risk and low-risk groups of TCGA, so we got a lower 37% to divide the patients into two groups. And for the CGGA data set, we used the same formula and percentile inherited from the TCGA data set. All the Time-dependent ROC were performed by the R package “survival ROC” (11). In this study, we construct a nomogram based on the risk score groups and clinical traits by the multivariable Cox regression analysis. Subsequently, validations were performed by discrimination and calibration using the R package “rms”. Concordance index (C‐index) was applied to calculate the discrimination of the nomogram. Then we evaluated the nomogram between the prediction probabilities and the observed rates using the calibration curves. The GSEA was performed by the R package “cluster profile” [22455463] (12–14).

## Results

### Selection of Candidate Genes to Build the Predictive Model

**Figure 1:**
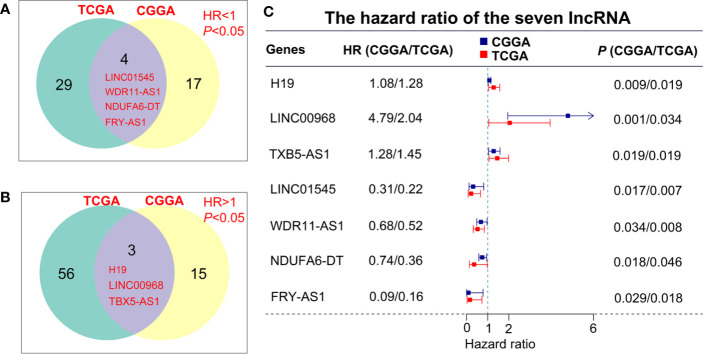
lncRNA screening in the TCGA and CGGA data sets. **(A, B)** The overlapping genes with HR>1 or HR<1 between TCGA and CGGA data sets. **(C)** Univariate cox regression analysis of 7 lncRNAs in the overall survival assessment.

**Figure 2:**
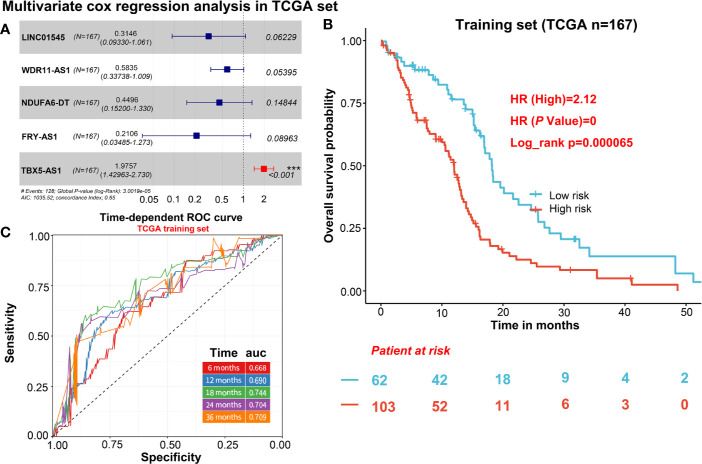
The selection of 5 candidate lncRNAs and prognostic capacity assessment. **(A)** 5 lncRNAs were selected *via* the multivariate cox regression analysis. **(B)** K-M OS curve plotting with the 5-lncRNA signature. **(C)** The prognostic capacity evaluation of the model built by 5-lncRNA signature.

To describe our research clearly, we draw a flow chart of the analysis procedure (**Figure S1**). Firstly, we selected the common genes of the TCGA and CGGA data sets, including 930 common lncRNAs. Then, we overlapped the genes with HR>1 of TCGA and CGGA, or HR<1 of TCGA and CGGA (**Figures 1A, B**). And we show the result of the 7 lncRNAs univariate cox regression analysis in **Figure 1C**. As the results show, the 7 lncRNAs were significantly correlated with the patient’s OS in both TCGA and CGGA. And evaluated H19, LNC00968, and TBX5-AS1 expressions were negatively correlated with the patient’s OS, LNC01545, WDR11-AS1, NDUFA6-DT, and FRY-AS1 were in reverse. Then we used the 7 lncRNAs to perform the multivariate cox regression analysis (**Figure 2A**) and got the 5-lncRNA signature with C-index 0.65 (**Figure 2A**). We use the 5-lncRNA signature to divide the patients into two groups based on the optimal cut-off value with 37% low-risk group. The K-M OS curve shows that the high-risk score was significantly correlated with lower OS (HR = 2.12, *P* < 0.0001; **Figure 2B**). The AUC of a time-dependent ROC curve was calculated to represent the prognostic capacity of the 5-lncRNA signature (**Figure 2C**). The AUCs of the 5-lncRNA biomarker prognostic model was 0.67, 0.69, 0.74, 0.70, and 0.70 for the 6, 12, 18, 24, and 36 months OS times, respectively, implicating that the model possesses certain accuracy and classification capability (**Figure 2C**).

### Evaluation the Predictive Model in Validation Cohorts

**Figure 3:**
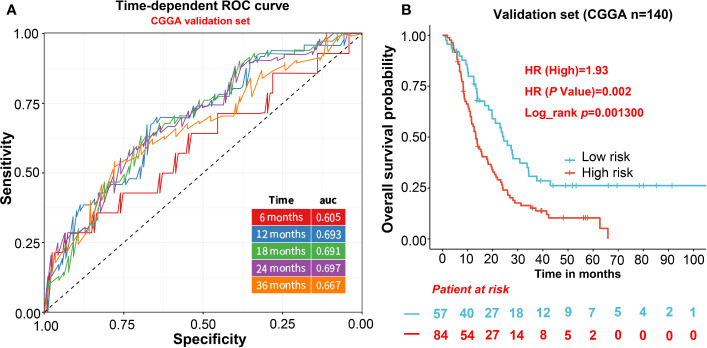
The evaluation of the predictive model in validation cohorts. **(A)** AUCs of time-dependent ROC curves for CGGA cohort. **(B)** The survival analysis of the CGGA validation cohort.

For independent validation, we assessed the predictive model in CGGA including 140 GBM samples. The risk score of CGGA was calculated in the same way as the TCGA data set. For the survival analysis of the CGGA validation cohort, we also calculated the risk score and divided patients into two groups based on a lower 37% same with TCGA. The result shows the high-risk score was significantly correlated with lower OS in the CGGA cohort (HR = 1.93, p = 0.0013; **Figure 3B**). Furthermore, the AUCs of time-dependent ROC curves for CGGA cohort were 0.61, 0.69, 0.69, 0.70, and 0.67 at 6, 12, 18, 24, and 36 months (**Figure 3A**), indicating that the predictive model had capacities for predicting OS in the GBM patients (**Figure 3A**).

### The Five-lncRNAs Signature Was Independent of Clinical Factors

The independent prognostic value of the five-lncRNAs signature was assessed by univariate and multivariate cox regression in 167 TCGA GBM with complete clinical information. As the result of **Table 1** shows, age, risk score group, radiation therapy, chemotherapy, and IDH status were correlated with the patient’s OS, and the risk score group of C-index value was almost reached to the radiation therapy and chemotherapy. Then we incorporated the risk score group and all clinical factors to perform the multivariate cox regression. And we find age, radiation therapy, and risk score group were the independent prognostic factors correlated with OS (**Table 1**). And the risk score group combined with the clinical factors model can increase the C-index from 0.604 to 0.736 (Δ = 0.132), compared to a multivariate model which was mainly based on clinical features (**Table 1**). In conclusion, the risk score was a significant independent predictor of BCR, indicating that it may contribute to the guidance of treatment decisions in the clinical practice.

**Table 1:** Multivariate cox regression of prognostic clinical factors.

Variable	Characteristics	Univariable Cox				Multivariable Cox				
		HR	95% CI	Pr (>|z|)	C-index	HR	95% CI	Pr (>|z|)	C-index	
**Age**	**Continuous**	1.03	1.01 to 1.04	0	0.603	1.02	1 to 1.04	0.05	0.736	
**Sex**	**Female** *vs* **Male**	1.07	0.81 to 1.4	0.636	0.514	0.74	0.5 to 1.1	0.132		
**Riskscore**	**High** *vs* **Low**	2.08	1.58 to 2.74	0	0.621	2.97	2.04 to 4.33	0		
**Radiation**	**Yes** *vs* **No**	0.28	0.2 to 0.4	0	0.628	0.26	0.14 to 0.47	0		0.604
**MGMT**	**Methylated** *vs* **Unmethylated**	0.78	0.6 to 1.02	0.073	0.546	–	–	–		
**Chemo**	**Yes** *vs* **No**	0.44	0.32 to 0.61	0	0.623	–	–	–		
**IDH1**	**Mutant** *vs* **Wildtype**	0.48	0.26 to 0.86	0.013	0.54	–	–	–		
**KPS**	**<70** *vs* **>=70**	0.76	0.54 to 1.06	0.108	0.552	–	–	–		

### Build the Risk Score Combined With Clinical Factors Nomogram

**Figure 4:**
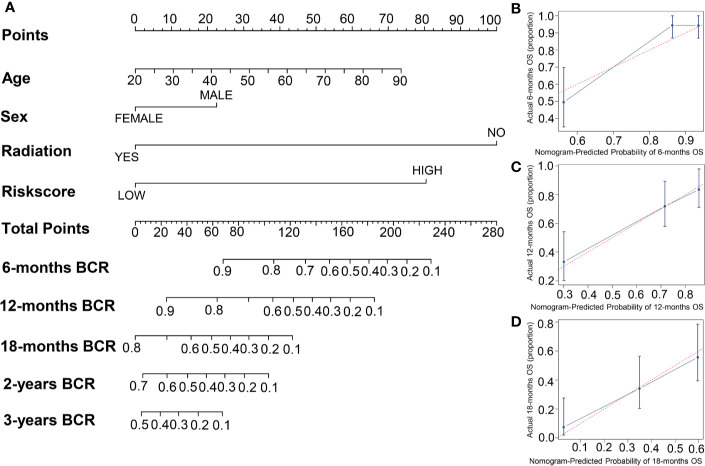
The building of a nomogram and its performance on the OS prediction. **(A)** A nomogram was built with four prognostic factors. **(B–D)** The OS-predicting performance of the nomogram was evaluated by calibration plots.

To establish a new model for predicting the patients’ OS in GBM, a nomogram was generated to predict the probability of the 6‐, 12‐, and 18‐months OS in the TCGA cohort. As **Table 1** has shown that age, sex, risk score, and radiation therapy were independent prognostic factors (Selecting criteria: significance threshold of log-rank *P <*0.05), they were therefore embodied in the nomogram (**Figure 4A**). And the nomogram was visualized with calibration plots. The actual OS was represented by the red line and the blue line represented the predictive OS. Calibration plots showed that the nomogram performed well (**Figures 4B–D**). And C-index was used to evaluate the combined model (C-index: 0.736), compared to the clinical model (C-index: 0.604) (**Table 1**).

### Interaction Analysis of Target Proteins and Enrichment of Tumor-Related Signaling Pathway for the Five-lncRNAs Signature

In order to confirm the accuracy of gene screening, we predicted the target proteins of the 5 lncRNAs (**Tables S1**-**S3**), and conducted an interaction network analysis of the proteins (**Figure S2**). Finding that TBX5-AS1 may be related to some oncogenic protein (15–18), while the other 4 lncRNAs may be associated to some tumor suppressor proteins (19–22). Indicating that these lncRNAs may be key genes in GBM. We also performed the differential expression genes (DEGs) analysis using the R package “limma”, and selected the DEGs (adjust. p value <0.05) to perform GSEA using the R package “cluster profile” and Wiki-pathway. The four most significant pathways were showed in **Figure S3** and **Table S4**. The risk group system was accompanied by PI3K-Akt, VEGFA-VEGFR2, TGF-beta, Notch, T-Cell Antigen Receptor signaling pathways, and so on (**Figure S3** **and** **Table S4**). And these pathways are significantly correlated with tumor progression. Although few lncRNAs have been functionally annotated in GBM, we revealed the relevant signaling pathways of the five lncRNAs through GSEA. Which proved the reliability and distinguishing capability of the formula.

## Discussion

LncRNA has been widely recognized as regulators for biological processes related to tumorigenesis and progression in GBM. Jiang et al. stated that blocking Lnc00152 can suppresses glioblastoma malignancy by impairing mesenchymal phenotype through the miR-612/AKT2/NF-κB pathway (23). Lnc-TALC has been proved to be associated with temozolomide (TMZ) resistance induced by AKT signaling pathway in GBM (24). Lnc-SChLAP1 can promote the growth of GBM through stabilization of ACTN4 and activation of NF-κB signaling (25). Growing evidence suggests that lncRNAs can be promising biomarkers for GBM diagnosis and treatment.

However, it is unconvincing to use a single lncRNA as a target due to the significant heterogeneity of GBM (26). More recently, the molecular research and gene exploration of the tumor, especially the statistical analysis research based on big data, has made rapid progress (27, 28). Polygenic research has become the focus of this field. In this present study, after comprehensive analysis of gene expression in GBM from TCGA and CGGA databases, five lncRNAs (LNC01545, WDR11-AS1, NDUFA6-DT, FRY-AS1, TBX5-AS1) were noticed abnormally expressed in GBM, which were also found related with OS. The five-lncRNAs signature-derived risk score presented satisfactory efficacies in predicting the therapeutic efficacy and prognosis in GBM. Additionally, high-risk score was enriched with items of signaling pathways for oncogenesis and tumor progression (29, 30).

The discovered DEGs might be more stably and specifically expressed in GBM based on TCGA and CCGA datasets (including 930 common lncRNAs), compared to just one dataset. Then univariate and multivariate Cox regression analysis were conducted on the DEGs, and five important genes were found associated with survival independently. Results show that TBX5-AS1 expressions were negatively correlated with the patient’s OS, which may function as oncogenes, whereas LNC01545, WDR11-AS1, NDUFA6-DT, and FRY-AS1 were in reverse, which may function as suppressor gene. A risk score model was then established on the basis of the signature of these five DEGs. Studies have indicated that gene signature containing multiple gene components can be more robust and convictive in the prediction of prognosis, while the usage of some single gene may result in instability and predictive bias. In the present study, the risk score based on the five-lncRNAs signature was effective and reproducible in predicting prognosis of the GBM patients from both TCGA and CCGA datasets. Additionally, the risk score model could also predict the radiotherapy response of GBM patient. Generally, the multi-gene signature-derived risk score model may be promising and valid in treatment evaluation and prognostic prediction in GBM.

LncRNA can promote or inhibit GBM by regulating signal transduction pathways, forming sponge adsorption effect, regulating the characteristics of glioma stem cells, regulating hypoxia response, and angiogenesis (31–33). The potential functions of the five genes may partially contribute to the prognostic prediction, but the underlying mechanisms upon these genes in GBM remains to be investigated (34–36). Of the five lncRNAs, apart from TBX5-AS1, which has been to reported be related with unfavorable prognosis in non-small-cell lung carcinoma (NSCLC) (37, 38), the other four has never been explored in cancer, and none of them has ever been explored in GBM. In this case, we believe that it is promising to explore the potential mechanisms of these genes (especially the TBX5-AS1 which acts as a risk factor in our model) related to GBM.

Generally speaking, clinical and pathological classification determine the treatment and prognosis of GBM. Independent of the traditional method, the value of this model lies in its clinical guidance for therapeutic strategies, which may help improve the prognosis in GBM patients. Intensive therapy should be applied to the patients with high-risk scores, while those with low risk scores should avoid excessive treatments that may cause therapeutic toxicities and deterioration. Although the values of AUCs for five genes were close to or more than 0.7, the accuracy and classification capability of the model are still not high enough, the interaction analysis of target proteins and enrichment of tumor-related signaling pathway proved the accuracy of gene screening and the reliability and distinguishing capability of the model. This also enlightens us to make some in-depth exploration on the mechanisms in the further study. On the other hand, compared with those genes that have just been screened and have not yet been verified, it may be another reliable choice to build models based on recognized genes. Moreover, except for TCGA, CGGA, and GEO, it is highly recommended to introduced other databases (e.g. NoncoRNA) in our further studies (39). Despite a long way ahead for the clinical application of this model, the prospects are considerable and worthy of further exploration.

## Conclusions

In conclusion, the five-lncRNAs signature-derived risk score presented satisfactory efficacies in predicting the therapeutic efficacy and prognosis in GBM and will be significant for guiding therapeutic strategies and research direction for GBM. Since most of the genes we put forward have not been detailedly researched, confirmatory experiments are necessary in the further studies.

## Data Availability Statement

Publicly available datasets were analyzed in this study. This data can be found here: The Cancer Genome Atlas (TCGA, https://cancergenome.nih.gov), and Chinese Glioma Genome Atlas (CGGA, http://www.cgga.org.cn/index.jsp).

## Author Contributions

XN, JS, and HL designed the study. LM, TF, and TZ collated the data. XN and JJ conducted data analyses and drafted the manuscript. All authors contributed to the article and approved the submitted version.

## Funding

This work was supported by Tianjin Major Science and Technology Projects (17ZXMFSY00200), Tianjin Science and Technology Development Strategy Research Projects (18ZLZXZF00740).

## Conflict of Interest

The authors declare that the research was conducted in the absence of any commercial or financial relationships that could be construed as a potential conflict of interest.

## Supplementary Material

Supplementary Figure 1 The flow chart of the analysis procedure for this study.

Supplementary Figure 2 Figure S2 Interaction network analysis of the target proteins for the five lncRNAs. The red circles represent the up-regulated proteins and the green represent the down regulated proteins.

Supplementary Figure 3 Identification of 5-lncRNAs signature associated biological signaling pathway. PI3K-Akt, VEGFA-VEGFR2, TGF-beta, Notch, T-Cell Antigen Receptor signaling pathways were the main enrichment pathways.

Supplementary Table 1 Prediction of target proteins for FRY-AS1, LNC01545, WDR11-AS1, and NDUFA6-DT.

Supplementary Table 2 Prediction of target proteins for TBX5-AS1.

Supplementary Table 3 Target protein prediction and related biological effects of the five lncRNAs.

Supplementary Table 4 The related signaling pathways of the 5-lncRNAs signature.

## Abbreviations

GBM, Glioblastoma; TCGA, The Cancer Genome Atlas; CGGA, Chinese Glioma Genome Atlas; LncRNA, Long non-coding RNA; DEGs, Differentially expressed genes; GSEA, Gene set enrichment analysis; OS, Overall survival.
