# Supplementary figures and images for: A Five-lncRNAs Signature-Derived Risk Score Based on TCGA and CGGA for Glioblastoma: Potential Prospects for Treatment Evaluation and Prognostic Prediction
Source: Front Oncol. 2020 Dec 17;10:590352. doi: 10.3389/fonc.2020.590352 (PMC7773845; doi:10.3389/fonc.2020.590352)

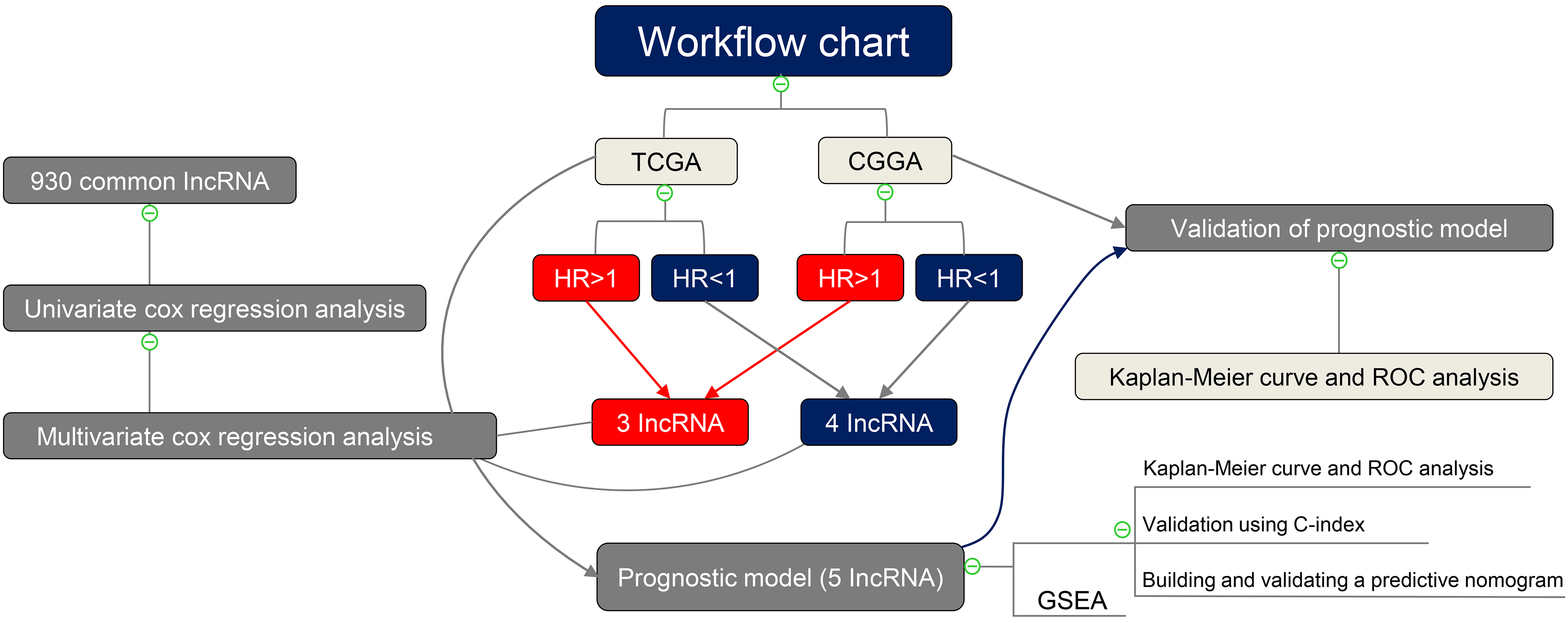

Supplement: Supplementary Figure 1 — The flow chart of the analysis procedure for this study. [file Image_1.tif]

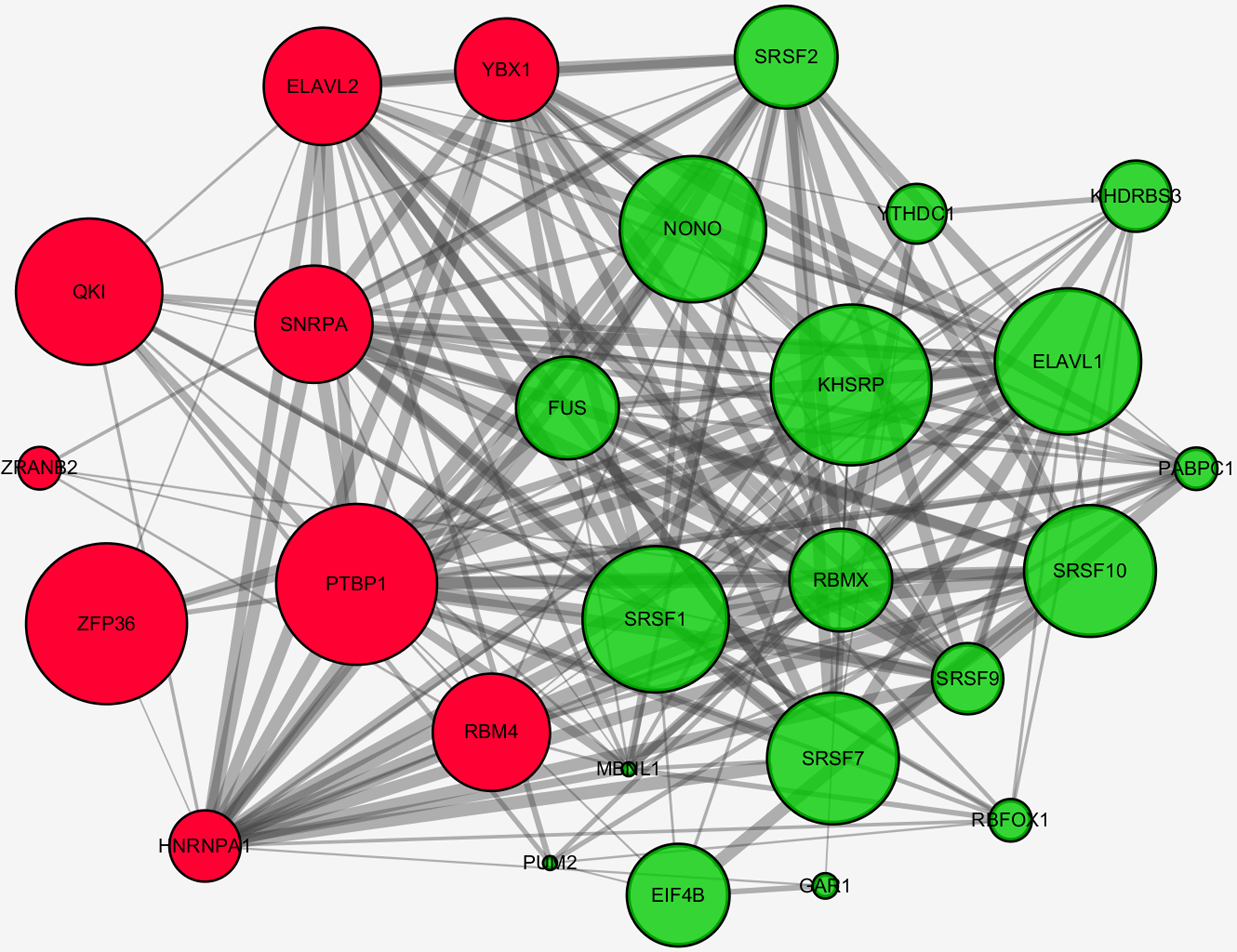

Supplement: Supplementary Figure 2 — Figure S2 Interaction network analysis of the target proteins for the five lncRNAs. The red circles represent the up-regulated proteins and the green represent the down regulated proteins. [file Image_2.tif]

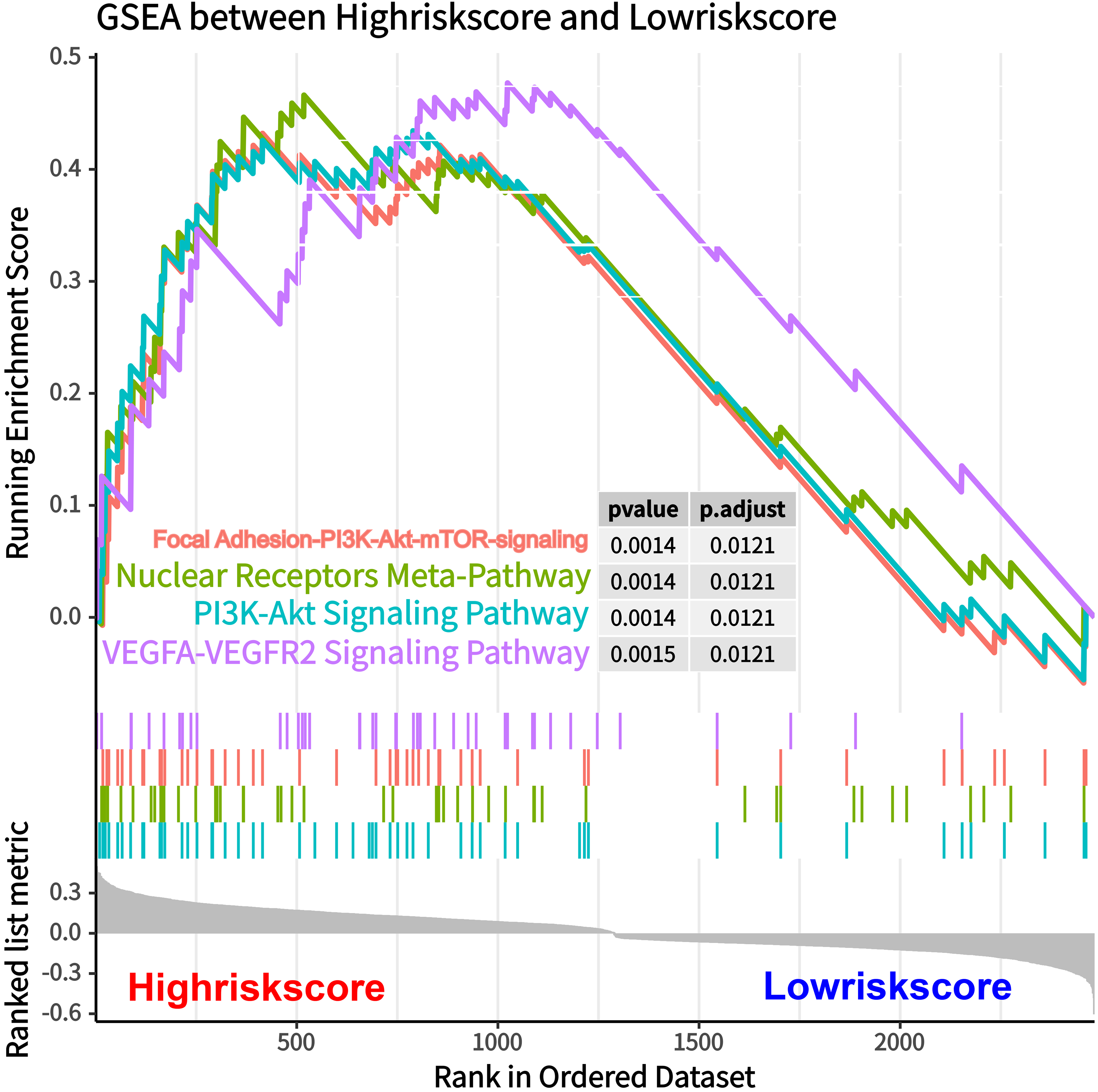

Supplement: Supplementary Figure 3 — Identification of 5-lncRNAs signature associated biological signaling pathway. PI3K-Akt, VEGFA-VEGFR2, TGF-beta, Notch, T-Cell Antigen Receptor signaling pathways were the main enrichment pathways. [file Image_3.tif]
