# Supplementary material for: A Five-lncRNAs Signature-Derived Risk Score Based on TCGA and CGGA for Glioblastoma: Potential Prospects for Treatment Evaluation and Prognostic Prediction
Source: Front Oncol. 2020 Dec 17;10:590352. doi: 10.3389/fonc.2020.590352 (PMC7773845; doi:10.3389/fonc.2020.590352)
Supplement: Supplementary Table 3 — Target protein prediction and related biological effects of the five lncRNAs. [file DataSheet_3.docx]

**Table S3. Target protein prediction and related biological effects of the five lncRNAs.**

| LncRNA | Target protein | Involved pathways | Biological effects | Reference |  |
| --- | --- | --- | --- | --- | --- |
| FRY-AS1 LNC01545 WDR11-AS1 NDUFA6-DT | NONO↓ | Nitric oxide/cGMP | Proliferation & migration↓ | 15 |  |
|  |  |  |  |  |  |
|  | KHSRP↓ | —— | Migration↓ | 16 |  |
|  |  |  |  |  |  |
|  | EIF-4B↓ | PI3K/AKT/mTOR | GBM cell death | 17 |  |
|  |  |  |  |  |  |
|  | ELAVL1↓ | —— | Protein synthesis↓ | 18 |  |
|  |  |  |  |  |  |
| TBX5-AS1 | PTBP↑ | USP5 alternative RNA splicing | Cell growth and migration↑ | 19 |  |
|  |  |  |  |  |  |
|  | ELAVL2↑ | —— | GBM progression↑ | 20 |  |
|  |  |  |  |  |  |
|  | QKI↑ | SHH/GLI1 | Cell stemness maintaining↑ | 21 |  |
|  |  |  |  |  |  |
|  | YBX1↑ | —— | Cell invasion↑ | 22 |  |
|  |  |  |  |  |  |
